# Development and validation of the thyroid cancer self-perceived discrimination scale to identify patients at high risk for psychological problems

**DOI:** 10.3389/fonc.2023.1182821

**Published:** 2023-07-18

**Authors:** Zhi-jin Liu, Lin-sen Feng, Feng Li, Li-rong Yang, Wan-qi Wang, Yuan He, Zong-ting Meng, Yu-feng Wang

**Affiliations:** ^1^ Department of Geriatric Oncology, The Third Affiliated Hospital of Kunming Medical University - Yunnan Cancer Hospital, Kunming, China; ^2^ Department of Hematology, The Sixth Affiliated Hospital of Kunming Medical University-Yuxi People’s Hospital, Yuxi, China; ^3^ Department of Hematology, General Medical College of Kunming Medical University, Kunming, China

**Keywords:** thyroid cancer, psychometrics, psycho-oncology, scale development, validation, quality of life

## Abstract

**Objective:**

To develop a Thyroid Cancer Self-Perceived Discrimination Scale (TCSPDS) to identify patients at high risk for psychological problems and to test its reliability, validity and acceptability.

**Methods:**

Using classical test theory, a total of 176 thyroid cancer patients from November 2021 to October 2022 were recruited to develop the TCSPDS. Item analysis was used to improve the preliminary TCSPDS. Exploratory factor analysis (EFA), confirmatory factor analysis (CFA) and structural equation model (SEM) were used to test the construct validity of the final TCSPDS. Pearson correlation coefficient was used to analyze the validity coefficient between TCSPDS and EORTC QLQ-C30 to test the criterion-related validity (CRV) of the final TCSPDS. The internal consistency coefficient (Cronbach’s alpha coefficient), split half reliability (Spearman-Brown coefficient) and test-retest reliability were used to verify the reliability of the final TCSPDS. The questionnaire completion time and effective response rate were used to validate the acceptability of the final TCSPDS.

**Results:**

The TCSPDS consisted of 20 items and was divided into 3 subscales: 8 items for stigma, 6 items for self-deprecation, and 6 items for social avoidance. The TCSPDS had good validity (χ^2^/df=1.971, RMSEA=0.074, GFI=0.921, CFI= 0.930, IFI=0.932, TLI=0.901, Validity coefficient=0.767), reliability (Cronbach’s alpha=0.867, Spearman-Brown coefficient=0.828, test-retest reliability coefficient=0.981) and acceptability [average completion time (15.01 ± 1.348 minutes) and an effective response rate of 95.14%]. Patients with higher TCSPDS scores reported a lower quality of life (*P*<0.05).

**Conclusion:**

The TCSPDS could be used for early identification and assessment of the level of self-perceived discrimination in patients with thyroid cancer, which may provide a scientific basis for health education, social support and psychosocial oncology services in the future, especially in Southwest China.

## Introduction

Over the past 30 years, the incidence of thyroid cancer has continued to rise globally (1). Data from the National Cancer Center of China in 2022 showed that the incidence of thyroid cancer in China was the second most common cancer among people aged 15 to 44 years (2). Among malignant tumors in women, thyroid cancer is the third most common cancer after breast cancer and lung cancer, and it seriously threatens the physical and mental health and socio-economic development of the nation (2).

“Discrimination” is a kind of psychological reaction and behavior among different social groups, including stigmatization, negation, exclusion and other unequal treatment in speech and behavior (3). “Self-perceived discrimination” refers to the intermittent or persistent negative psychological state of a member of a special population after subjectively predicting or objectively experiencing negative, exclusionist and other unfair social behaviors of other social groups, which includes shame, stigma, fear, anxiety and depression (4, 5). However, most studies on disease-related self-perceived discrimination mainly focus on patients with infectious diseases, mental disorders and disabilities (6–8). There are few studies on cancer-related self-perceived discrimination, especially thyroid cancer (9, 10).

With improvements in the treatment effect and life expectancy of thyroid cancer patients, cancer-related psychological problems have gradually become the new focus and have received global attention. Although thyroid cancer, which is mainly treated with surgery, is regarded as a “good cancer”, the risk of treatment toxicity (include neck wounds, thyroid dysfunction, paresthesia, pain, etc.) and long-term recurrence, as well as the continuous negative effects on social and family relationships, result in widespread and nonnegligible self-perceived discrimination among patients and significantly affect their quality of life (QOL) (11–13).

Previous studies have shown that self-perceived discrimination of thyroid cancer patients may come from cancer phobia, treatment methods and economic burden. The public’s fear of cancer stems from the fact that cancer is a malignant disease that is difficult to cure and eventually leads to the patient’s death (14). Patients newly diagnosed with thyroid cancer still show obvious fear and anxiety about disease recurrence and death even after receiving health education with good prognosis (15). Second, patients often felt pain and helplessness related to the change in appearance after surgery, which was more obvious within 6 months; the appearance of injury in female patients may be particularly serious (16, 17). Furthermore, the continuous increase in treatment costs and the decline in work ability undoubtedly caused a heavy economic burden for patients. The objective economic burden usually developed into the self-perceived burden of patients, which may be more prominent in low-income patients (18, 19).

At present, there is no suitable tool specifically designed to assess self-perceived discrimination in thyroid cancer patients. Only the Shame and Stigma Scale (SSS), developed by DW Kissane et al. for patients with head and neck cancer, is currently available (20). The SSS contains four subscales: shame with appearance, regret, sense of stigma and speech/social concerns. Although the SSS had good validity and reliability, it focused on oral cancer and could only evaluate the patient’s perceived stigma, which is only one component of self-perceived discrimination. Moreover, although our previously developed Cancer Self-Perceived Discrimination Scale (CSPDS) could be used to preliminarily estimate the self-perceived discrimination of all cancer patients, it still lacks specificity for thyroid cancer (21).

Therefore, the purpose of our study was to develop and validate a specific Thyroid Cancer Self-Perceived Discrimination Scale (TCSPDS) to identify patients at high risk for psychological problems, and analyze the correlation between TCSPDS and QOL.

## Methods

### Study design and participants

Based on classical test theory, the steps were as follows:

(1) A preliminary TCSPDS was developed through a literature review, semi-structured interviews, expert consultations and cognitive interviews.
(2) A questionnaire was formed to investigate patients with thyroid cancer.
(3) The final TCSPDS was formed by the item analysis method.
(4) The final TCSPDS was validated.
(5) The independent influencing factor of TCSPDS was analyzed.
(6) The correlation between TCSPDS and QOL was analyzed.

Patients with thyroid cancer treated at Yunnan Cancer Hospital were selected as the research objects. Individuals who were patients from November to December 2021 were selected as the semi-structured interview objects, and those from March to October 2022 were selected as the questionnaire objects.

The inclusion criteria for patients included the following:

(1) had a diagnosis of thyroid cancer by histopathology and thyroidectomy was performed (tumor staging followed AJCC Version 8 criteria) (22),
(2) had good language expression capability and understanding ability,
(3) were aware of their diagnosis and volunteered to participate in the study, and
(4) were ≥18 years old.

The exclusion criteria for patients were as follows:

(1) had other primary cancers, or
(2) had infectious diseases, mental disorder, brain trauma history, physical disability or underwent emergency medical treatment.

This was a single-center study with all participants from the Yunnan Cancer Hospital, Yunnan Province, China. Ethical approval for this study was granted by the Ethics Committee of Yunnan Cancer Hospital (NO. KYLX2022063). All participants signed an informed consent form. We promised the participants that the data would only be used for this study, and that the participants may withdraw from the study at any time.

### Development of the preliminary TCSPDS

#### Step 1

The random sampling method was used to select thyroid cancer patients who could reflect the target population of the research questions to the greatest extent for semi-structured interviews (from November 1 to December 20, 2021). Before the interview, qualitative research experts were consulted to help understand the necessary precautions for the interview process, and relevant training was conducted for researchers to ensure the reliability of the interview data. An interview outline was established by reviewing a large number of studies: (1)How did you feel psychologically after being diagnosed with thyroid cancer? (2) Did your disease affect your life? Was there any psychological burden? (3)Had you ever been treated unfairly? How was it handled? (4) Were you cared for by others? (5) How have your relationships with your family and friends changed? Were you willing to share your diagnosis with them? When necessary, the researchers asked additional follow-up questions based on the patient’s answers.

With the consent of the participants, the researchers introduced the research purpose, methods and privacy protection measures to them. Individual interviews were conducted in the inpatient talk room, and general information such as age, sex, marital status, education level, treatment methods, and disease duration were collected. Participants were informed that the interview content would be recorded in the form of an audio recording or notetaking during the interview, and they were assured that the interview content would only be used for this research study and would not be released. Each participant was interviewed for approximately 35 to 45 minutes, and their real names were replaced by numbers.

The study followed the principle of the “information saturation method”, so that the sample size of participants depended on whether further information was available in the semi-structured interview (23). The interview data were sorted out within 24 hours after the end of the interview. Based on the construction grounded theory, two researchers applied NVivo 12 PLUS software to conducted independent selective coding on the interview data to extract the theme and subtheme, and a third researcher made decisions when disagreements occurred.

#### Step 2

The literature related to self-perceived discrimination in cancer patients was reviewed, and an item pool was established through semi-structured interviews and literature reviews (20, 21, 24–28). The language design (grammar and sentence patterns) should refer to the literature review and conform to Chinese grammar (from November 18 to December 25, 2021). Items were expressed on a 5-point Likert scale, and for the forward-scored items, the options were as follows: 1=strongly disagree, 2=somewhat disagree, 3=not sure, 4=somewhat agree, and 5=strongly agree. For the reverse-scored items, the score was reversed. Therefore, a higher score indicated a higher level of self-perceived discrimination.

#### Step 3

The Delphi expert consultation method was used to invite 4 experts in medical oncology, 2 experts in head and neck surgery and 1 expert in linguistics to evaluate the content and applicability of the items in two rounds (from January 3 to February 8, 2022) (29). In the first round of expert consultation, the inappropriate descriptions were removed, and the expressions of the items were further revised. In the second round of expert consultation, a 4-point rating scale (1=uncorrelation, 2=weak correlation, 3=moderate correlation, 4=strong correlation) was used to independently review and assess each item by experts, and the item-level content validity index (I-CVI) was calculated. If any item had an I-CVI<0.80, we would repeat the above steps until the item had an I-CVI≥0.80, or it would be deleted.

#### Step 4

Ten patients with thyroid cancer were invited to give cognitive interviews on the content and meaning of each item to ensure that all items could be understood and accepted in the formal investigation (from February 10 to February 28, 2022). For each item, 10 participants answered the question “Can you understand and accept the content and meaning of the item?” Finally, the preliminary TCSPDS was formed.

### Questionnaire development and distribution

The questionnaire consisted of three parts: (1) The general information questionnaire included age, sex, marital status, residential area, economic burden, education level, disease duration and operation method. (2) The preliminary TCSPDS was included. (3) The European Organization for Research and Treatment of Cancer Quality of Life Questionnaire (EORTC QLQ⁃C30) was included (30). The EORTC QLQ⁃C30 has been widely used to evaluate the QOL of cancer patients. It covered 15 domains with 30 items, including 5 functional domains, 9 symptom domains and one global health domain. Its Cronbach’s alpha was 0.884, which indicated good reliability and validity. Items 1 to 28 from EORTC QLQ⁃C30 were scored on a 4-point scale, with scores from 1 to 4 indicating no, somewhat, fairly or very much. Items 29 and 30 were rated from 1 to 7 based on patient responses, with higher scores indicating lower QOL.

For factor analysis, the common recommendation for sample size was N≥100, and the ratio between independent and dependent variables was at least 5 (31, 32). Before administration of the survey, the researchers were trained professionally. Questionnaires were distributed to patients with thyroid cancer who came to the hospital for treatment and were collected on site. In principle, the questionnaire should be completed by patients according to their own assumptions. However, it could be completed under the guidance of researchers, if necessary. The formal investigation was conducted from March 1 to October 20, 2022.

### Validation of TCSPDS

#### Step 1: item analysis

The Pearson correlation coefficient was used to analyze the correlation between each item’s score and the total score of the scale. If the Pearson correlation coefficient of any item was less than 0.3 or was not significant (*P*>0.05), then it was deleted.

#### Step 2: validity analysis

The I-CVI was used to verify the content validity of the TCSPDS. If the I-CVI>0.8, the content validity of the scale was considered to be good. Exploratory factor analysis (EFA), confirmatory factor analysis (CFA) and structural equation modeling (SEM) were used to verify the construct validity of the TCSPDS. First, EFA of SPSS 25.0 software was used to construct the theoretical model. If the Kaiser−Meyer−Olkin (KMO) was greater than 0.7 and the significance of Bartlett’s test was less than 0.001, the common factor was considered suitable to for extraction (33). Then, CFA and SEM of AMOS 24.0 software was applied to verify the theoretical model. If the criteria were met for the chi-square degree of freedom ratio (χ^2^/df<2), root mean square error of approximation (RMSEA<0.08), goodness-of-fit index (GFI>0.90), comparative fit index (CFI>0.90), normed fit index (NFI>0.90), incremental fit index (IFI>0.90) and Tucker−Lewis index (TLI>0.90), then the scale could be considered to have good construct validity (34). Pearson correlation coefficient was used to analyze the validity coefficient between TCSPDS and EORTC QLQ-C30 to test the criterion-related validity (CRV) of TCSPDS. If the validity coefficient *r*≥0.50 (*P*<0.05), the CRV was considered to be good (35).

#### Step 3: reliability analysis

The Cronbach’s alpha coefficient, Spearman-Brown coefficient and test-retest reliability were used to verify the reliability of the TCSPDS. If the Cronbach’s alpha and Spearman-Brown coefficients were at least 0.7, then it was considered to have good reliability (36). The intraclass correlation coefficient (ICC) was used to verify the test-retest reliability of the TCSPDS. If the ICC was at least 0.8, then it was considered to have good test-retest reliability (37).

#### Step 4: acceptability analysis

The acceptability of the questionnaire was verified by the effective response rate and average completion time. The questionnaire was considered to have good acceptability if the effective response rate was at least 90% and the average completion time was at most 20 minutes (38).

### Application of TCSPDS

#### Step 1: influencing factors analysis

One-way ANOVA was used to compare the scores of TCSPDS among different participants, and multiple stepwise regression analysis was used to analyze the independent influencing factors of patients’ TCSPDS scores.

#### Step 2: QOL correlation analysis

The Pearson correlation coefficient was used to analyze the correlation between the scores of TCSPDS and QOL. If the correlation coefficient *r*<0.4 (*P*<0.05), it indicated that the correlation was weak; If 0.4≤*r*<0.7 (*P*<0.05), it indicated that the correlation was moderate; If *r≥*0.7 (*P*<0.05), it indicated that the correlation was strong (39).

SPSS 25.0, AMOS 24.0 and NVivo 12 PLUS were used for statistical analysis. Two-tailed *P*<0.05 was considered significant.

## Results

### Participant characteristics

A total of 185 patients with thyroid cancer agreed to participate, and 176 patients completed the survey (effective response rate, 95.14%). Among the 176 participants, 141 were female and 35 were male; 105 were younger than 45 years old and 71 were older than or equal to 45 years old. Their disease duration was as follows: 86 had disease duration less than or equal to 6 months and 90 had disease duration more than 6 months. Detailed information about the participants’ characteristics is shown in **Table 1**.

**Table 1:** Participants characteristics( $\bar{\text{χ}}$ ± s).

Characteristics		Number (%)	TCSPDS score	*F*	*P*
Sex				6.183	0.024
	Male	35 (19.89)	57.26±6.68		
	Female	141 (80.11)	61.09±9.36		
Age				2.552	0.000
	<45 year	105 (59.66)	58.10±8.92		
	*≥*45 year	71 (40.34)	63.63±8.13		
National				0.881	0.247
	Ethnic Han	145(82.39)	59.97±8.78		
	Ethnic Minorities	31(17.61)	62.03±10.01		
Marital status				2.208	0.113
	Married	147 (83.52)	60.68±8.83		
	Unmarried	21 (11.93)	56.76±7.27		
	Divorced or widowed	8 (4.55)	63.25±14.24		
Residence area				0.885	0.000
	Rural area	59 (33.52)	64.47±8.13		
	Urban area	117 (66.48)	58.24±8.74		
Monthly income				2.982	0.000
	<5000 RMB	59(33.52)	65.78±7.48		
	*≥*5000 RMB	117(66.48)	57.58±8.47		
Medical insurance				12.642	0.000
	Employee	70(39.77)	57.30±8.47		
	Urban resident	45(25.57)	59.24±9.12		
	NCMS	61(34.66)	64.61±7.95		
Economic burden				0.554	0.000
	Light	90 (51.14)	54.86±7.17		
	Heavy	86 (48.86)	66.06±6.96		
Education level				3.333	0.000
	Junior high school and below	74 (42.05)	64.88±7.46		
	Senior high school and above	102 (57.95)	57.03±8.62		
Tumor stage				7.736	0.000
	I	157(89.20)	59.96±8.66		
	II	4(2.27)	47.50±5.80		
	III	13(7.39)	66.23±7.79		
	IV	2(1.14)	77.00±1.41		
Disease duration				3.325	0.000
	≤6 month	86 (48.86)	62.76±9.52		
	>6 month	90 (51.14)	58.01±7.87		
Operation method				15.392	0.000
	Endoscopic thyroidectomy	33 (18.75)	62.80±7.99		
	Open thyroidectomy	143 (81.25)	49.61±3.84		

### Development of the preliminary TCSPDS

According to the in-depth semi-structured interviews, the three themes of stigma, self-deprecation and social avoidance were extracted from the self-perceived discrimination of 35 thyroid cancer patients. The stigma included cancer fear, wound and voice changes, irritable temper and medication labels. Self-deprecation included dysfunction, disfigurement and financial burden. Social avoidance included disease concealment, communication disorder and refusal of care.

Based on the qualitative research and literature review, a preliminary TCSPDS with 12 items was developed. In the first round of expert consultation, we revised three items. We revised item 3 “Some people believe that thyroid cancer is a genetic disease” to “Some people believe that thyroid cancer is inherited”. Item 11, “I hate seeing myself in the mirror”, was revised to “Sometimes I feel distressed about my neck wound”. Item 13, “Sometimes I feel responsible for my thyroid cancer”, was revised to “Sometimes I feel very guilty about my thyroid cancer”. In the second round of expert consultation, the I-CVIs of the 12 items were all greater than 0.80. After cognitive interviews with 10 thyroid cancer patients, no revision of the item content was needed.

### Validation of TCSPDS

#### Item analysis

The Pearson correlation coefficient between the score of each item and the total score of the TCSPDS ranged from 0.343 to 0.722 (*P*<0.01). Therefore, there was no need to remove any item from the TCSPDS.

#### Content validity

After two rounds of expert consultation, the I-CVIs of all 12 items in the preliminary TCSPDS were greater than 0.80. Each item was strongly correlated with the total scale, and Pearson correlation coefficients ranged from 0.343 to 0.722 (*P*<0.01). Each item was strongly correlated with each subscale, and Pearson correlation coefficients ranged from 0.472 to 0.874 (*P*<0.01). Therefore, it was determined that the TCSPDS had good content validity.

#### Construct validity

The EFA results showed that the KMO of the preliminary TCSPDS was 0.802, and Bartlett’s test was 695.088 (*P*<0.001), which indicated that it was suitable for factor analysis. Three common factors with characteristic roots greater than 1 were extracted by principal component analysis (PCA), and the cumulative variance contribution rate was 56.525%. The detailed factor loadings are shown in **Table 2**.

**Table 2:** Preliminary TCSPDS factor loadings (n=176).

Items	*r*	Common factors		
		F1	F2	F3
8. Sometimes I'm dissatisfied with the appearance of my neck.	0.855	**0.914**	0.135	0.037
11. Sometimes I feel distressed about my neck wound.	0.779	**0.881**	0.043	0.033
15. I don't like others staring at my neck.	0.772	**0.876**	0.061	0.038
9. The change of appearance made me lose confidence and attraction.	0.565	**0.626**	0.269	0.317
1. Some people will avoid me because of my wound.	0.644	**0.593**	0.083	0.534
3. Some people believe that thyroid cancer is inherited.	0.394	**0.451**	0.208	-0.384
4. Sometimes I feel my temper has become irritable.	0.561	0.005	**0.749**	0.033
7. Taking medicine is my label.	0.515	0.271	**0.617**	-0.248
12. My sleep and memory became worse after the operation.	0.398	0.073	**0.612**	0.134
13. Sometimes I feel very guilty about my thyroid cancer.	0.340	0.107	**0.532**	0.213
16. I don't like going to public place.	0.569	-0.061	0.296	**0.691**
2. My hoarse voice makes others feel uncomfortable.	0.391	0.178	-0.001	**0.600**
Characteristic value		3.450	1.833	1.500
Cumulative variance contribution rate (%)		28.749	44.023	56.525

The meaning of the bold values was that the factor loadings were greater than 0.40.

According to the qualitative research results, Factor 1 was named stigma, which mainly reflects the fact that thyroid cancer patients were treated unfairly because of their disease and disfigurement. Factor 2 was named self-deprecation, which mainly reflected the negative psychology of patients under multiple burdens, such as dysfunction, economic, social and psychological burdens. Factor 3 was named social avoidance, which mainly reflected the actual condition of disease concealment, communication disorder and refusal of care.

**Figure 1:**
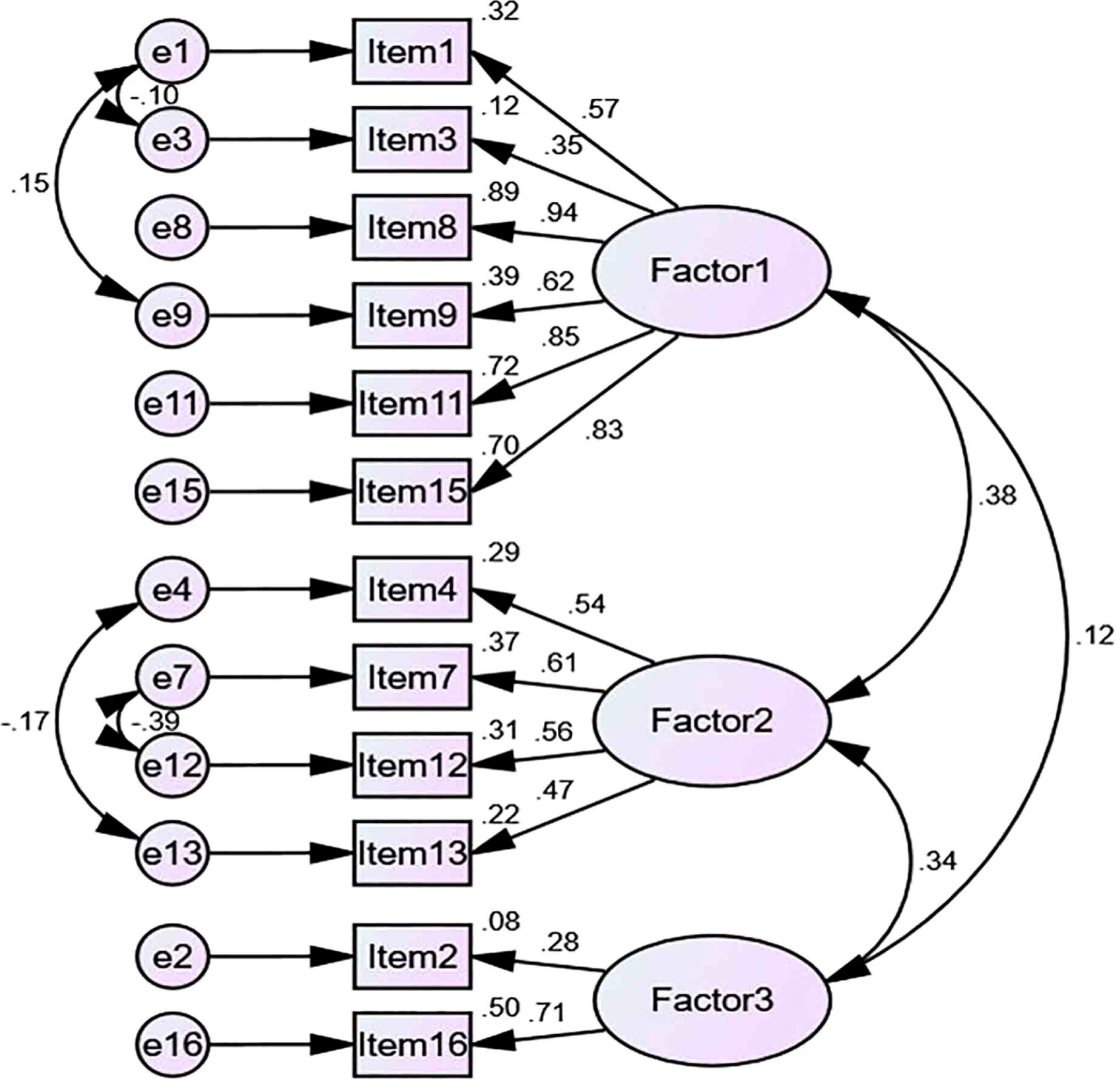
Structural Equation Model of TCSPDS.

According to the theoretical model constructed by EFA, CFA and SEM were conducted on the distribution of each item of the scale. The results showed that the fitting degree of the correlated three-factor model was better than that of the uncorrelated three-factor model. However, the NFI was slightly lower than the threshold, and the other fitting index performed well, which indicated that the TCSPDS had good construct validity (**Figure 1** and **Table 3**). The 8 items previously verified in the CSPDS were added to the corresponding subscale of the TCSPDS, and these 8 items were universal to all cancer patients (20). Finally, the final TCSPDS with 20 items was formed. Except for item 20, which was a reverse item, all the items were forward items (**Table 4**).

**Table 3:** Fitting index of confirmatory factor analysis for TCSPDS (n=176).

Factor structure	*χ^2^/df*	*RMSEA*	G*FI*	*CFI*	*NFI*	*IFI*	*TLI*
Uncorrelated three-factor model	2.261	0.085	0.901	0.901	0.839	0.903	0.872
Correlated three-factor model	1.971	0.074	0.921	0.930	0.870	0.932	0.901
Threshold	<2.00	<0.08	>0.90	>0.90	>0.90	>0.90	>0.90

**Table 4:** The final TCSPDS.

Items	Factors
1. Some people will avoid me because of my wound.	F1
2. My hoarse voice makes others feel uncomfortable.	F3
3. Some people believe that thyroid cancer is inherited.	F1
4. Sometimes I feel my temper has become irritable.	F2
5. People look down upon thyroid cancer patients.	F1
6. Some people avoid me for fear that I will borrow money from them	F1
7. Taking medicine is my label.	F2
8. Sometimes I'm dissatisfied with the appearance of my neck.	F1
9. The change of appearance made me lose confidence and attraction.	F1
10. Sometimes, I feel I am useless.	F2
11. Sometimes I feel distressed about my neck wound.	F1
12. My sleep and memory became worse after the operation.	F2
13. Sometimes I feel very guilty about my thyroid cancer.	F2
14. Sometimes I feel like a burden to my family.	F2
15. I don't like others staring at my neck.	F1
16. I don't like going to public place.	F3
17. If too many people know about my condition, it could be bad for me.	F3
18. Thyroid cancer means fewer and fewer friends.	F3
19. I hate others talking about my disease behind me.	F3
20. I like others to visit and care about me.	F3

F1, Stigma; F2, Self-deprecation; F3, Social avoidance.

#### Criterion-related validity

The validity coefficients of TCSPDS, shame subscale, self-deprecation subscale and social avoidance subscale with EORTC QLQ-C30 were 0.767, 0.585, 0.761 and 0.617 respectively (*r*≥0.50, *P*<0.01), which indicated that the TCSPDS had good CRV (**Table 5**).

**Table 5:** Correlation between TCSPDS and QOL.

	TCSPDS	Stigma	Self-deprecation	Social avoidance
QOL	0.767^b^	0.585^b^	0.761^b^	0.617^b^
Physical function	0.467^b^	0.335^b^	0.407^b^	0.468^b^
Role function	0.550^b^	0.414^b^	0.420^b^	0.582^b^
Emotional function	0.516^b^	0.348^b^	0.595^b^	0.404^b^
Cognitive function	0.262^b^	0.177^a^	0.423^b^	0.088
Social function	0.603^b^	0.476^b^	0.498^b^	0.554^b^
Fatigue	0.341^b^	0.166^a^	0.349^b^	0.430^b^
Nausea/vomiting	0.115	0.103	0.117	0.046
Pain	0.559^b^	0.465^b^	0.389^b^	0.557^b^
Global health	0.651^b^	0.469^b^	0.617^b^	0.602^b^
Dyspnoea	0.327^b^	0.202^b^	0.261^b^	0.410^b^
Insomnia	0.284^b^	0.192^a^	0.457^b^	0.097
Appetite loss	0.177^a^	0.152^a^	0.089	0.204^b^
Constipation	0.155^a^	0.122	0.232^b^	0.037
Diarrhea	0.040	0.060	0.040	-0.018
Financial difficulties	0.509^b^	0.465^b^	0.456^b^	0.320^b^

Pearson correlation coefficient: ^a^ P<0.05, ^b^ P<0.01.

#### Reliability

The Cronbach’s alpha of the final TCSPDS was 0.867, and the Spearman-Brown coefficient was 0.828. The Cronbach’s alpha values of the three subscales were 0.850, 0.650 and 0.628, and the Spearman-Brown coefficients were 0.741, 0.671 and 0.641. These results indicated that the final TCSPDS had good internal reliability. Two weeks after the first questionnaire survey, 14 patients with thyroid cancer participated in the second survey. The test-retest results showed that the ICCs of the final TCSPDS and the three subscales were 0.981, 0.976, 0.861 and 0.967, which indicated that the final TCSPDS had good test-retest reliability.

#### Acceptability

The average completion time of the TCSPDS with 20 items was 15.01 ± 1.348 (range: 13 to 19) minutes. A total of 185 final TCSPDS questionnaires were distributed, and 176 valid questionnaires were recovered (effective response rate was 95.14%), which indicated that the final TCSPDS had good acceptability.

### Application of TCSPDS

#### Influencing factors of TCSPDS

One-way ANOVA indicated, statistically significant differences in scores of sex, age, area of residence, monthly income, type of medical insurance, economic burden, education level, tumor stage, disease duration and operation method (*P*<0.05), as shown in **Table 1**. TCSPDS scores were used as dependent variables, and the above 10 statistically significant variables were used as independent variables. Through multiple stepwise regression analysis, the results showed that gender, economic burden, disease duration and operation method were independent influencing factors of patients’ self-perceived discrimination (*P*<0.05), as shown in **Table 6**.

**Table 6:** The independent influencing factors of TCSPDS.

Projects	Regression coefficient	Standard error	Normalized regression coefficient	*t*	*P*
Sex	5.797	1.032	0.258	5.618	0.000
Economic burden	7.150	0.882	0.398	8.108	0.000
Disease duration	-5.052	0.829	-0.281	-6.097	0.000
Operation method	-11.681	1.143	-0.508	-10.224	0.000

#### QOL correlation analysis

The scores of TCSPDS were positively correlated with the scores of QOL, global health, 5 functional domains and 7 symptom domains (fatigue, pain, dyspnea, insomnia, appetite loss, constipation and financial difficulties) (*P*<0.05), and there was a strong correlation between TCSPDS and QOL (*r*=0.767, *P*<0.01). These results indicated that patients with higher TCSPDS scores reported a lower QOL, poorer global health and function, and more prominent symptoms (**Table 5**).

## Discussion

Our study showed that the self-perceived discrimination of thyroid cancer patients was mainly manifested in three dimensions: stigma, self-deprecation and social avoidance. Although patients had objective stigma from others, patients’ subjective self-deprecation and social avoidance were more common. Therefore, we developed a 20-item TCSPDS based on classical test theory specifically to assess self-perceived discrimination in thyroid cancer patients. Exploratory factor analysis extracted three common factors: stigma, self-deprecation and social avoidance. The correlated three-factor model had a good fit with the initial theory (χ^2^/df=1.971, GFI=0.921, CFI=0.930, TLI=0.901). The I-CVI, the content correlation (Pearson correlation coefficients ranged from 0.343 to 0.722, *P*<0.01) and the validity coefficient (*r*=0.767, *P*<0.01) were satisfactory. These results proved that the TCSPDS had good content, construct and criterion-related validity. The Cronbach’s alpha of the TCSPDS was 0.867, the Spearman-Brown coefficient was 0.828, and the ICC was 0.981, which proved that the TCSPDS had good reliability. The completion time (15.01 ± 1.348 minutes) and effective response rate (95.14%) of the TCSPDS were satisfactory, which suggested that the TCSPDS had good acceptability. Thus, the TCSPDS can be used to estimate the self-perceived discrimination factors of stigma, self-deprecation, and social avoidance associated with thyroid cancer.

Compared with the CSPDS and SSS, the TCSPDS has higher reliability and comprehensiveness. The reliability of the TCSPDS was higher than that of the CSPDS (Cronbach’s alpha=0.829) (21). Although the TCSPDS had eight items that were similar to items on the CSPDS, we included 12 items that were more comprehensive in assessing self-perceived discrimination of thyroid cancer, such as disfigurement and glandular dysfunction. Furthermore, although the Cronbach’s alpha of the TCSPDS may be lower than that of the SSS (Cronbach’s alpha=0.930) (20), the TCSPDS had better construct validity and test-retest reliability, and we added a self-deprecation subscale. Most thyroid cancer patients said in the interview that self-deprecation was the main cause of self-perceived discrimination, so adding the self-deprecation subscale may make the evaluation content more comprehensive.

The specificity of TCSPDS is higher than that of CSPDS and SSS. The CSPDS is a universal scale for all cancer patients (21), which can only preliminarily evaluate the self-perceived discrimination shared by all cancer patients but cannot estimate the self-perceived discrimination specific to thyroid cancer, so it still lacks specificity. The SSS mainly focuses on the influence of the facial appearance of patients with oral cancer (20), while the biological characteristics, treatment methods and prognosis of thyroid cancer are different from those of oral cancer; in particular, the implementation of endoscopic thyroidectomy has greatly improved the appearance of the neck of patients with thyroid cancer. Therefore, the TCSPDS may be a more promising early screening tool for self-perceived discrimination in thyroid cancer.

In China, because of the lack of health knowledge, the public is afraid of “talking about cancer”. Some people believe in feudal superstitions and mistakenly believe that cancer is “retribution from heaven” and even think that being close to cancer patients will lead to “misfortune”, and thus deliberately avoid cancer patients. The change in appearance may reduce confidence for thyroid cancer patients, causing them to fear communicating with others face to face or going out in public, as they want to avoid others seeing their neck wound. Thyroid cancer patients can be very embarrassed if others pay attention to their neck wounds. Therefore, they usually try to avoid the sight of others by lowering their heads or pulling up their collars. However, owing to the lack of thyroid gland function after surgery, long-term oral administration of “levothyroxine sodium tablets” has undoubtedly become a characteristic label for patients with thyroid cancer. Moreover, patients usually need their families to accompany them to their medical treatments, yet they cannot provide help in return to their families. Due to the internal pressure of family responsibility, patients tend to think that they are a burden to their families. Consequently, our research provides more evidence for cancer-related discrimination.

Interestingly, our study found that financial burden, sex, disease duration and operation method were independent risk factors for patients’ self-perceived discrimination. Patients with heavy economic burdens have higher self-perceived discrimination, and continuously increasing costs of thyroid cancer treatment and long-term review, and a decline in physical fitness and work ability caused by the toxicity of the treatment is less conducive to their return to society, which undoubtedly brings a heavy economic burden to their entire families one that often aggravates their self-perception burden (18). In addition, patients with short disease duration and open thyroidectomy had stronger self-perceived discrimination, which may be related to postoperative toxicity, such as physiological and social dysfunction and image disfigurement, especially for female patients. Patients often have difficulty adapting to their sudden change in appearance after an open thyroidectomy, and a poor appearance is more likely to cause others’ rejection and aversion, undoubtedly having a heavy psychological blow on female patients (40). Although endoscopic thyroidectomy has certain advantages, a large proportion of patients still undergo open thyroidectomy for various reasons, such as the presence of lateral neck compartment lymph node metastasis, local invasive carcinoma, previous neck surgery or irradiation, and financial difficulties. Therefore, more attention needs to be paid to the mental health status of open thyroidectomy patients (41). However, as time passes, patients’ self-perceived discrimination may improve to some extent from the positive psychological adjustment effect, which can help them recover their original mental state (42). Therefore, in addition to paying attention to the treatment of primary diseases, medical staff should also pay more attention to patients’ psychological problems, and actively prevent and alleviate their self-perceived discrimination, to reduce the occurrence of mental diseases.

Notably, the TCSPDS is negatively correlated with QOL in patients with thyroid cancer, and there is a strong correlation between the two (*r*=0.767, *P* < 0.01). Patients with higher TCSPDS scores have lower QOL, worse global health and function, and more prominent symptoms. Although thyroid cancer has a good prognosis, long-term psychological distress, such as disease recurrence and fear of death and dysfunction, may make it difficult for patients to realize their own social values and maintain stable interpersonal relationships (11–13, 43). Self-perceived discrimination may aggravate the antagonism and contradiction between thyroid cancer patients and the general population, and the problems of disfigurement and long-term dysphonia further affect the normal social activities of patients (44, 45). Additionally, the insomnia, fatigue and paresthesia caused by treatment toxicity not only aggravate patients’ physical decline and feelings of inferiority but also significantly affect patients’ daily life and work (46–48). Therefore, the TCSPDS will help with early identification of these problems and will help the development and implementation of targeted health education, psychological counseling and social support. This may ultimately improve the QOL in patients with thyroid cancer.

As an exploratory study, this study had a limited sample size and was derived from a single center. The sample used in this study only included people from Southwest China, where economic development is relatively underdeveloped, so the results of this study only represent the situation of thyroid cancer patients in Southwest China. Future research should cover a larger sample size of multiple centers and multiple fields to analyze the correlation between self-perceived discrimination and other negative psychological factors (anxiety, depression, fear, etc.). Further exploration of the impact mechanism of self-perceived discrimination on the QOL will provide more powerful evidence to reduce the self-perceived discrimination of thyroid cancer patients and improve their QOL.

If any researcher needs to use this scale, we allow him or her to translate it into other languages or make modifications and confirm its validity and reliability.

## Conclusion

Our study shows that the TCSPDS is effective and reliable in assessing self-perceived discrimination in thyroid cancer patients and can be used as a basis for health education, psychological counseling and social support in the future, especially in Southwest China.

## Data availability statement

The original contributions presented in the study are included in the article/supplementary material. Further inquiries can be directed to the corresponding author.

## Ethics statement

The studies involving human participants were reviewed and approved by Ethics Committee of Yunnan Cancer Hospital. The patients/participants provided their written informed consent to participate in this study. Written informed consent was obtained from the individual(s) for the publication of any potentially identifiable images or data included in this article.

## Author contributions

Z-JL: Study design, data analysis and manuscript preparation. L-SF, FL, L-RY, W-QW, YH, Z-TM: Data collection. Y-FW: Study design, data interpretation and manuscript modification. All authors read and approved the final manuscript.
